# Predictors of social networking service addiction

**DOI:** 10.1038/s41598-023-43796-2

**Published:** 2023-10-04

**Authors:** Hyeon Jo, Eun-Mi Baek

**Affiliations:** 1 Headquarters, HJ Institute of Technology and Management, Jungdong-ro 71 39, Bucheon-si, Gyeonggi-do 14721 Republic of Korea; 2 Department of Preventive Medicine, College of Medicine, Catholic University of Korea, Seoul, Republic of Korea

**Keywords:** Psychology, Mathematics and computing

## Abstract

The surge in social network services (SNS) usage has ignited concerns about potential addictive behaviors stemming from excessive engagement. This research focuses on pinpointing the primary determinants of SNS addiction by introducing a theoretical framework centered on flow, perceived enjoyment, and habit. A sample of 282 SNS users from South Korea was surveyed, and the gathered data was assessed through partial least squares structural equation modeling (PLS-SEM). The evaluation revealed that positive affect closely relates to flow and perceived enjoyment, whereas negative affect amplifies flow but diminishes perceived enjoyment. Additionally, the research underscored that social influence significantly shapes habits and affects perceived enjoyment. Notably, flow demonstrated a strong connection to addiction, and perceived enjoyment influenced both flow and habit significantly. Habit was directly linked to addiction. These insights pave the way for more in-depth studies on SNS addiction patterns and offer a foundation for devising effective strategies to mitigate its adverse effects.

## Introduction

With the rapid proliferation of social network services (SNS), users' daily routines have evolved significantly^1–3^. SNS provides insights into acquaintances' updates, new product launches, and current events^4–6^. Given SNS's profound influence, users are dedicating more time to these platforms^7^. Among various tools, SNS apps are most frequented on smart devices^8^. Some users develop a habitual pattern of SNS usage, which, in extreme cases, turns addictive^9,10^. SNS addiction can be defined as an excessive, compulsive use of social media platforms that interferes with daily life, leading to negative consequences in physical, social, and mental well-being^11^. It involves an increased craving to engage on these platforms, leading to the neglect of offline relationships and daily responsibilities^12^. Ironically, this addiction can overshadow the genuine connections that SNSs aim to cultivate. Numerous studies have explored the variables driving SNS user behavior^13,14^, attributing psychological elements, social influencers, and predisposition to SNS addiction^15–17^. However, there remains a gap in comprehensively understanding social and psychological influences on addiction. This research seeks to bridge that gap by examining user affect, social stimuli, and mental states.

The rise in SNS over-reliance or addiction is a modern behavioral addiction that alarms researchers and mental health experts alike^11^. This trend is notably prevalent among university students, a major user group. Analyzing SNS addiction determinants among this group is crucial for multiple reasons. Firstly, high SNS engagement in students correlates with adverse psychological outcomes like depression, anxiety, and loneliness^18,19^. It also interferes with sleep and hampers academic success^20^. Thus, comprehending SNS addiction's roots can help alleviate these issues. Furthermore, university students are in a crucial life phase, establishing habits that might extend into their later life^21^. Recognizing and addressing addictive patterns during this period can circumvent future repercussions^22^. As a dominant SNS user group, understanding students' addiction can enable tailored intervention strategies. Hence, exploring SNS addiction's drivers among university students can advance mental health, foster efficient interventions, and deepen our grasp on behavioral addiction.

The dual factor model of Facebook use^23^ posits that individuals use SNS to manage both positive and negative affects. Positive affect boosts user satisfaction and flow in SNS^24^. Users in a good mood can lose track of time on SNS, further enhancing their addictive tendencies. Negative affect, denoting distressing emotions^25^, also impacts SNS flow^24^. Those with high negative affect might use SNS reflexively to counter negative feelings. This behavior aligns with the manifestation of habits^26^. Higher negative affect can diminish enjoyment levels. Users might engage with these platforms to amplify positive feelings or mitigate negative ones. If such strategies become compulsive, they might foster SNS addiction. Moreover, individuals with pronounced negative affect might be susceptible to behavioral addictions like SNS addiction^27^, using them as coping mechanisms. While positive affect usually brings beneficial outcomes, in some contexts, it can contribute to SNS addiction. If SNS consistently evokes positive feelings, it might reinforce and lead to an addiction cycle. Some research highlights a positive correlation between positive affect and addictive behaviors^28^. Considering both affects allows a thorough study of the emotional aspects of SNS addiction among university students. These insights can guide the creation of interventions targeting addictive SNS behaviors.

Social influence represents the degree to which a person's attitudes, beliefs, and behaviors are affected by others^29^. Users who are highly influenced by acquaintances will use SNS more frequently. Users who use SNS more tend to become addicted to SNS^30^. Thus, users with a higher level of social influence may be more prone to SNS addiction. Users who hear a lot about SNS from their acquaintances may be more immersed in using SNS than those who do not. They may also habitually use SNS to check the influence of their surroundings. Because SNS essentially forms fun and motivation to use via social relationships, social influence may increase perceived enjoyment. Additionally, this study investigates the impacts of negative affect on addiction, flow, habit, and perceived enjoyment.

Flow, a concept introduced by Csikszentmihalyi^31^, describes the state where one becomes deeply engrossed in an activity, losing all sense of time and self-awareness. Such flow significantly influences online users' addiction^32,33^. Regarding SNS, flow is viewed as an addiction precursor^34^. This immersion makes users neglect other priorities, encouraging addictive behaviors. Perceived enjoyment is another driver. As per self-determination theory^35^, inherent satisfaction from activities motivates behavior. Thus, students enjoying SNS may overuse, leading to addiction^33,36^. Perceived enjoyment also impacts flow^37,38^. Habit, marked by automatic responses and lack of intent^39^, also drives SNS addiction, especially among university students. Habitual SNS usage can be an automatic reflex to triggers like boredom, potentially escalating to addiction^40^. Habit strength, denoted by SNS usage frequency, is a predictor of addiction^10,30,41^. By studying flow, enjoyment, and habit, we obtain a holistic view of the psychological dynamics underlying SNS addiction.

The primary objective of this study is to comprehensively examine the multifaceted relationship between individual emotional responses (both positive and negative affects), social influences, flow experiences, perceived enjoyment, habitual behaviors, and the potential development of addiction towards SNS among university students. Through this exploration, the research aims to shed light on the underlying psychological and behavioral dynamics that may predispose individuals to SNS addiction, thereby offering insights into potential intervention and prevention strategies tailored to this demographic.

Although there is an extensive body of literature addressing the determinants of addiction to SNS, certain theoretical gaps remain unbridged. Firstly, the bulk of the research tends to position positive and negative affect on a singular continuum, rather than recognizing them as distinct constructs. This oversimplification potentially obscures the individual contributions of each affective state to SNS addiction. Secondly, the intricate relationships between positive and negative affect and other psychological determinants, including flow, perceived enjoyment, and habit, remain underexplored, particularly within the demographic of university students. This paper addresses the above gaps in the literature and contributes to the field in following ways. Firstly, it takes a novel approach by considering positive affect and negative affect as independent variables, allowing for a more nuanced understanding of their respective roles in SNS addiction. Secondly, it extends the current literature by exploring the interplay between positive affect, negative affect, social influence, flow, perceived enjoyment, and habit in the context of SNS addiction among university students. The conceptual foundation of our study is rooted in the dual factor model of SNS Use, which emphasizes the regulatory function of both positive and negative affects in SNS engagement^23^. Anchored by Deci and Ryan’s self-determination theory^35^, we propose that inherent satisfaction derived from activities becomes a potent motivator of behavior, especially in the context of SNS usage. Csikszentmihalyi’s theory of Flow also informs our study, positing that users become deeply engrossed in activities, losing awareness of time, which, in the context of SNS, can precipitate addiction. Collectively, this theoretical framework will guide our exploration of the psychological and behavioral nuances influencing SNS addiction among university students.

This paper delves into such an unresearched dimension, shedding light on the intricate interplay of flow, habits, and perceived enjoyment as drivers of SNS addiction. While extant literature has ventured into the domains of health, loneliness, and attachment in relation to SNS addiction, the unique combination of factors examined herein offers a fresh perspective. This underscores the originality of our research, marking a distinct departure from conventional narratives. By merging previously disjointed variables and unveiling their collective impact on SNS addiction, we not only bridge a significant gap in the current scholarship but also provide readers with a compelling rationale to delve deeper into our findings. In so doing, we aspire to catalyze further academic discourse and innovative research in this domain.

This article is structured as follows. Section “Literature review” describes the theoretical background. Section “Research model” delineates the research model and hypotheses. Section “Methodology” covers data and measurement tools. Section “Results” presents the analysis results of the measurement model and structural model. Section “Discussion” shows the discussion. Finally, Section “Conclusion” introduces a summary, implications, and limitations.

## Literature review

Over the past few decades, there has been a meteoric rise in the popularity and reach of SNS. This expansion has not only changed the way individuals communicate and interact but has also paved the way for a vast digital market. The ubiquitous nature of these platforms, combined with their design geared towards continuous engagement, has led to growing concerns among researchers, psychologists, and sociologists. The core of these concerns revolves around the potential addictive nature of these platforms. Given the profound impact of SNS on modern life, an increasing body of research has been dedicated to exploring the phenomenon of SNS addiction, its underlying causes, and its multifaceted implications on individual and societal well-being.

Affect, broadly categorized into positive and negative emotions, plays a pivotal role in determining how individuals interact with, perceive, and are influenced by SNS platforms. SNS addiction, characterized by excessive and compulsive use of SNS platforms despite negative repercussions^42^, has been closely tied to affective states. Positive affect often drives the 'reward-seeking' behavior, causing users to chase the dopamine rush associated with likes, comments, and social validation on these platforms^41^. Conversely, negative affect often results in escapism, where users resort to SNS to avoid or numb their negative feelings, eventually leading to addictive patterns^43^. Positive emotions have been linked to increased engagement with SNS platforms. Users in a positive mood state tend to share more, interact positively with others, and spend more time on SNS^44–46^. Furthermore, positive affect enhances the intrinsic motivation to use SNS, increasing the frequency and duration of usage^47^. There is evidence to suggest that people with high levels of positive affect use SNS as a medium to maintain and strengthen social connections, amplifying their feelings of social belonging and self-worth^48^. Contrarily, negative affect has a more nuanced relationship with SNS. While some research indicates that individuals experiencing negative emotions resort to SNS as a coping mechanism^49^, others argue that prolonged SNS use, especially passive browsing, can exacerbate negative emotions^50^. Moreover, the “social comparison theory” postulates that individuals with high levels of negative affect are more prone to compare themselves to others on SNS, which can amplify feelings of inadequacy and further deepen the negative emotional state^51^. In addition to affect, several studies have introduced attachments to explain SNS addiction. Monacis et al.^52^ assessed the psychometric properties of the Italian version of the Bergen Social Media Addiction Scale using confirmatory factor analysis. They evaluated five dimensions of adult attachment and clarified a theoretical relationship between SNS addiction and attachment style. Park and Oh^53^ identified the key factors influencing SNS addiction in pre-service teachers, finding that anxiety attachment and avoidant attachment significantly elicit SNS addiction. Furthermore, insecure adult attachment was found to mediate the effect of covert narcissism on SNS addiction.

Social influence, broadly defined, encompasses the array of ways in which individuals change their thoughts, feelings, and behaviors as a result of interacting with others^54^. Several studies have linked the role of social influence to increasing time spent on SNS platforms. Algorithms designed to show content from close connections or popular trends create a reinforcing loop, wherein users continuously engage to stay updated and relevant^55^. Furthermore, the witnessing of peers frequently engaging with or endorsing certain content or platforms can create a normative behavior pattern, leading individuals to subconsciously conform and potentially enter into a cycle of addictive behavior^56^. While social influence plays a significant role, the extent of its impact on an individual can vary based on personal factors. Those with low self-esteem or a high need for social validation may be more susceptible to SNS addiction under strong social influence^51^. Conversely, individuals with strong personal resilience and critical media literacy might navigate SNS spaces without succumbing to addictive behaviors, despite prevalent social influences. In summary, the intricate relationship between societal impact and dependency, particularly within the domain of SNSs, presents a diverse and intricate area of exploration. As society becomes increasingly digital, comprehending these intricacies becomes crucial in fostering constructive online conduct and lessening the vulnerabilities of dependency.

Scholars have addressed the concepts of flow, perceived enjoyment, and habit to explain behaviors associated with addiction to SNSs. Flow has been observed in various digital experiences, including gaming, website browsing, and SNS usage^57^. This deep immersion can amplify the appeal of SNS, making users more prone to spend extended periods in such platforms. Researchers like Faiola et al.^58^ have argued that achieving a state of flow can lead to repeated usage, which, over time, can contribute to addictive behaviors. This is because the gratification derived from such immersive experiences makes users more likely to seek them out repetitively. In the context of SNS, perceived enjoyment signifies the pleasure users derive from platform activities, be it scrolling, posting, or interacting with peers. Studies have identified perceived enjoyment as a strong predictor of SNS use^13,36^. Platforms that offer high levels of enjoyment can foster continued and increased usage. Over time, the search for this intrinsic pleasure can drive individuals to use SNS excessively. This compulsive need to seek out enjoyment can pave the way for addictive behavior patterns^11^. Furthermore, the daily ritual of checking and engaging with SNS can lead to the formation of strong habits^18^. With notifications and constant updates, SNS platforms are designed to encourage routine interactions, facilitating the transformation of casual usage into habitual behavior^59,60^. As habits become deeply ingrained, they can become automatic responses, often executed without much thought or conscious intention. This automaticity is concerning as users might find themselves compulsively checking SNS without any particular reason or prompt, signaling potential addiction^41^. Lee et al.^24^ posited that SNS addiction has a significant impact on flow and user satisfaction, finding that addiction positively affects flow. Gong et al.^61^ explored the factors affecting mobile SNS addiction, discovering that flow plays a crucial role in increasing the level of addiction. They also found that enjoyment, sociability, and informational value are major antecedents of flow, leading to a higher level of addiction. Seo and Ray^62^ examined the effects of habit and addiction in SNS use, revealing that immersion and concern for social acceptance are significant factors in increasing addictive use. Habitual use was also shown to have a positive influence on addictive use. Focused immersion is conceptually similar to flow in this study.

Additionally, researchers have studied SNS addiction by various perspectives. Yang et al.^10^ considered SNS addiction as pathological behavior in the context of mobile SNS, while high engagement was classified as non-pathological behavior. It was revealed that SNS enjoyment significantly impacts both addiction and high engagement, and that habit is significantly related to addiction. Osatuyi and Turel^30^ examined the precursors of SNS addiction symptoms using the dual system theory. They found that habit affects SNS addiction symptoms both directly and indirectly. Social self-regulation was also significantly associated with SNS addiction symptoms. Pontes et al.^63^ studied the role of cognitive-related factors and psychiatric distress in SNS addiction, demonstrating that fear of missing out, maladaptive cognitions, and psychiatric distress significantly contribute to addiction. Turel and Serenko^36^ summarized three theoretical perspectives: the cognitive-behavioral model, the social skill model, and the socio-cognitive model. Griffiths^64^ pointed out potential controversy over SNS addiction, suggesting improvements in methodological design, sample representativeness, and scale validity to bridge the gap between empirical findings.

Even with an abundance of studies focusing on these areas, a comprehensive exploration that seamlessly combines affect, social influence, flow, perceived enjoyment, and habit is noticeably absent. In light of this, our study endeavors to weave these constructs together, forging a cohesive blueprint that underscores their collective influence on SNS addiction. Through this endeavor, we aspire to deliver crucial insights to scholars and professionals alike, charting a path towards strategies that encourage healthier interactions with SNS platforms.

## Research model

**Figure 1:**
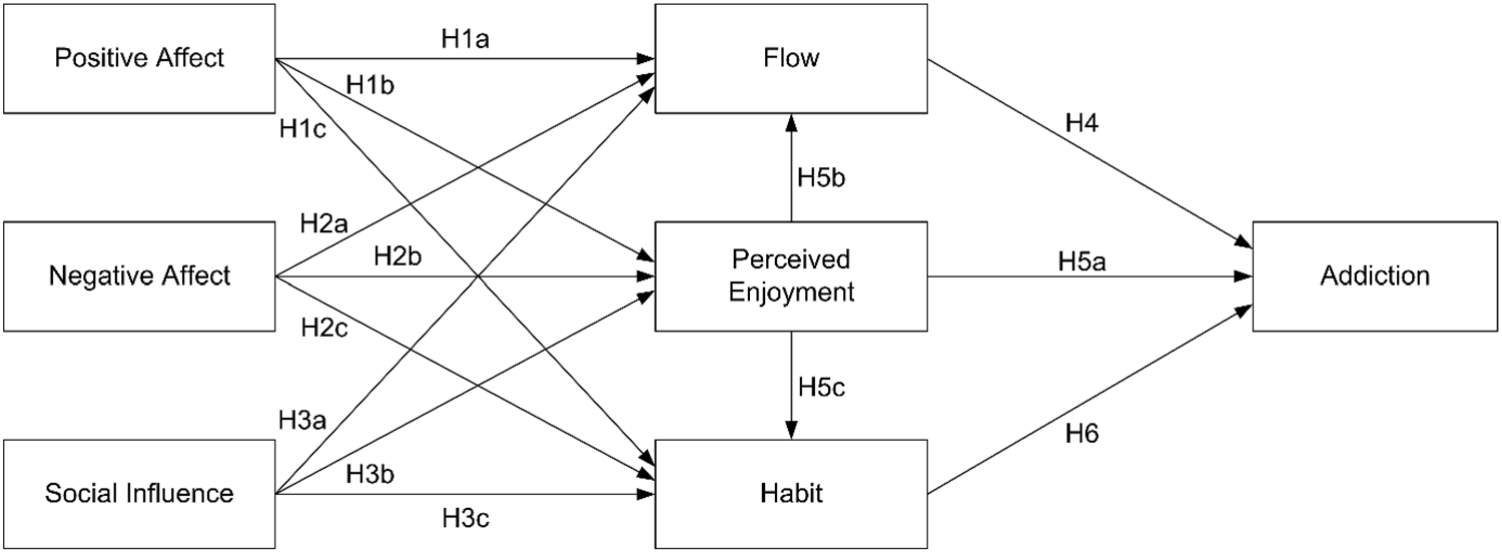
Research model.

Figure 1 presents the research model for understanding the determinants of SNS addiction. This study elucidates the roles of flow, perceived enjoyment, and habit in leading to addiction. It proposes that positive affect, negative affect, and social influence have significant impacts on the precursors of addiction.

### Positive affect

Positive affect, as defined by Watson et al.^25^, pertains to the extent to which individuals feel active, alert, and enthusiastic. Previous research suggests a significant relationship between positive affect and flow^65,66^ as well as perceived enjoyment^13^. In the context of SNS usage among college students, those with higher levels of positive affect may become more immersed in SNS activities to sustain these positive emotions, consequently exhibiting addictive behavior. They may experience a sense of joy due to their positive feelings and thus repetitively open the SNS app. Based on these observations, the current study posits that positive affect serves as a potent factor in the development of addiction, flow, habit, and perceived enjoyment.

#### H1a

Positive affect significantly influences flow.

#### H1b

Positive affect significantly influences perceived enjoyment.

#### H1c

Positive affect significantly influences habit.

### Negative affect

Negative affect is characterized as a state of distress and unenjoyable engagement that encompasses a range of aversive mood states^25^. In the context of SNS use among college students, negative affect can significantly influence their engagement with these platforms. As students experience higher levels of negative affect, they may alter their flow and habits on SNS platforms in an attempt to mitigate these uncomfortable feelings. For instance, students might increase their usage of SNS as a coping mechanism to distract themselves from their negative emotions^67,68^. Alternatively, they could also withdraw from SNS platforms due to their decreased enjoyment derived from the platforms when in a negative mood state. Consequently, negative affect can significantly influence the flow, habit formation, and perceived enjoyment associated with SNS use.

#### H2a

Negative affect significantly influences flow.

#### H2b

Negative affect significantly influences perceived enjoyment.

#### H2c

Negative affect significantly influences habit.

### Social influence

Social influence refers to the degree to which they are influenced by each other's actions in social relationships^69^. It includes relational norms and the identity that users feel like a member of society. Social influence significantly determines the intention to use SNS^70,71^. The ultimate purpose of users using SNS is to form and experience social relationships. In this context, users who are greatly influenced by their acquaintances may experience addiction and flow. The more people around users have an influence on SNS, the more users will try to use it repeatedly and enjoy it. Hence, social influence is believed to significantly affect addiction, flow, habit, and perceived enjoyment.

#### H3a

Social influence significantly influences flow.

#### H3b

Social influence significantly influences perceived enjoyment.

#### H3c

Social influence significantly influences habit.

### Flow

Flow refers to the holistic experience that individuals feel when they act with total involvement^72^. It significantly affects addiction in several online contexts^32,33^. Focused immersion is positively related to the addictive use of SNS^62^. Flow has a significant association with SNS addiction^61,73^. Thus, one can expect that flow serves as a crucial factor in shaping addiction.

#### H4

Flow significantly influences addiction.

### Perceived enjoyment

Perceived enjoyment is a key intrinsic motivation for information system usage^74^. It plays a preeminent role in enhancing addiction to online behavior^33^. Perceived pleasure was also found to be significantly related to social networking addiction^75–77^. The more SNS users enjoy social media activities, the more they would be immersed and immersed in them. SNS enjoyment serves as the salient factor in generating addiction and habit^10^. Given the above, this study is expected to show that perceived enjoyment significantly impacts addiction, flow, and habit.

#### H5a

Perceived enjoyment significantly influences addiction.

#### H5b

Perceived enjoyment significantly influences flow.

#### H5c

 Perceived enjoyment significantly influences habit.

### Habit

Habit represents repeated patterns of behavior that occur automatically without conscious awareness^78^. Habit significantly drives the experience of addiction symptoms^30^ and positively influences mobile SNS addiction^10^. Habitual use of SNS drives addictive use^62^. Hence, it is predicted that habit has a significant effect on addiction.

H6. Habit significantly influences addiction.

## Methodology

This study was approved by the Institutional Review Board (IRB) of HJ Institute of Technology and Management (HJITM), ethical committee of HJITM (HJITM-IRB-22-10-0008). In accordance with the ethical guidelines provided by the committee, we ensured to obtain in written form informed consent from all the study participants. All participants were informed about the purpose and the nature of the study, their rights to anonymity and confidentiality, and their freedom to withdraw from the study at any time without penalty.

### Subjects and data collection

This research focused on a specific demographic group, namely, full-time undergraduate students from various higher education institutions, due to their noted high engagement with SNS and the consequent potential for addictive behavior. Eligibility for participation in this study was based on the following criteria: participants were required to be currently enrolled full-time undergraduate students within the age range of 19–30 years. Furthermore, participants were required to exhibit active engagement with at least one Facebook, indicated by daily logins or frequent activity on the platform. Prospective participants failing to meet these established criteria were excluded from the study.

Facebook was selected as the platform of interest for this study due to several reasons. First, as of our knowledge cutoff in March 2023, Facebook remains one of the most popular and widely used SNSs globally, with billions of monthly active users^79^. This extensive user base increases the likelihood of obtaining a representative and diverse sample for the study, enhancing the external validity of our findings. Second, Facebook's comprehensive features, ranging from text updates, image and video sharing, livestreaming, private messaging, groups, events, to various interactive activities, make it a robust platform for studying diverse user behaviors. The variety of tools and features on Facebook may contribute to a higher risk of addictive behaviors as users have multiple avenues for engagement. Last, previous research on SNS addiction has frequently used Facebook as the platform for study due to its popularity and diverse user demographic. This consistency allows for easier comparison of results across different studies, thereby contributing to a more cohesive body of literature on SNS addiction.

In advance of participant recruitment, an a priori power analysis was conducted using DanielSoper calculation^80^. The results indicated a necessary minimum sample size of 200 participants for achieving an alpha level of 0.05 and a power of 0.80, thus ensuring the detection of medium-sized effects. In order to secure a representative sample, we employed a stratified sampling technique, taking care to ensure adequate representation from a variety of institutions, faculties, and academic years. An electronic questionnaire was disseminated through a range of online platforms and college forums, with a clear emphasis on informed consent and guaranteed anonymity of the respondents.

Data collection was carried out over a period of two months. During the distribution of the questionnaire, we collaborated with several professors who graciously assisted with the sampling, allowing us to target a diverse group of students from various majors and academic years. Additionally, we disseminated the survey through online communities and portals frequented by university students. By using these methods, we gathered data from a broad range of university student respondents. The instruments in questionnaire incorporated sections dedicated to the collection of demographic data, SNS usage patterns, and specific psychological variables of interest including affect, flow, habit, perceived enjoyment, and levels of SNS addiction. Following the collection of data, analysis was conducted using SPSS software. Descriptive statistics were initially employed to provide a summary overview of the collected data, with subsequent inferential statistical analyses performed to test the established research hypotheses. A total of 282 responses were used for the final analysis. Table 1 presents the demographic characteristics of the study's respondents. The total sample consisted of 282 participants. Regarding gender distribution, 127 participants (45.0%) identified as male, while 155 participants (55.0%) identified as female. This indicates a slightly higher representation of female respondents in the study. The age distribution among the respondents was grouped into three categories: those in their teens (10s), twenties (20s), and thirties (30s). A significant proportion of the participants fell within the teen and twenties age categories, with each constituting 32.3% and 35.8% of the total respondents respectively. Participants in their thirties made up the smallest age group, with 90 respondents (31.9%). It should be noted, however, that these percentages overlap due to the significant number of individuals in their early twenties who are also technically in their late teens. In summary, the respondent profile reflects a diverse and representative sample of individuals with varied demographic characteristics, which strengthens the generalizability of the study's findings.

**Table 1:** Profile of respondents.

Demographics	Item	Subjects (N = 282)	
		Frequency	Percentage (%)
Gender	Male	127	45.0
	Female	155	55.0
Age	10s	91	32.3
	20s	101	35.8
	30s	90	31.9

### Measurement instrument

Table 2 presents the definitions of the primary research variables utilized in the study. Positive affect and negative affect are defined based on the emotional reactions users experience when engaging with SNS, and they are sourced from Beatty and Ferrell^81^. Social influence represents the perception of the need to use SNS to stay current or due to recommendations, as identified by Li^71^. Flow, sourced from Gong et al.^61^, describes the immersive and singularly focused state of a user on SNS activities. Davis et al.^74^ provides the definition for perceived enjoyment, emphasizing the pleasure and interest users derive from SNS. Habit, according to Limayem et al.^26^, is characterized by a consistent and unconscious tendency to use SNS during leisure or to alleviate boredom. Lastly, Addiction captures the intense involvement in SNS, leading to diminished real-world social interactions and a decrease in positive emotions, as described by Osatuyi and Turel^30^.

**Table 2:** Definitions of research variables.

Variable name	Definition	Source
Positive affect	The feelings of excitement, passion, and pride experienced when using SNS	Beatty and Ferrell^81^
Negative affect	The feelings of suffering, anger, and annoyance experienced when using SNS	Beatty and Ferrell^81^
Social influence	The perception that using SNS is necessary to remain updated or due to external recommendations	Li^71^
Flow	The state of being completely immersed and focused solely on the activities of SNS	Gong et al.^61^
Perceived enjoyment	The feelings of pleasure, interest, and fun derived from using SNS	Davis et al.^74^
Habit	The regular, unconscious pattern of using SNS to kill time or relieve boredom	Limayem et al.^26^
Addiction	A state where users are deeply engrossed in SNS, resulting in reduced real-life social interactions and decreased positive emotions	Osatuyi and Turel^30^

In measuring constructs in this study, all questions were adapted from previously validated studies in the information systems and social networking services fields. These items were modified to fit the context of SNS use. All indicators were assessed using a seven-point Likert scale, ranging from 1 (strongly disagree) to 7 (strongly agree). The measurement of SNS addiction was done using self-reporting scales, comprising various items that gauge the frequency, intensity, and the negative implications of SNS use. In our research, we use the following items Osatuyi and Turel^30^: "I was immersed in SNS and experienced a decrease in conversations when meeting people.", "As I used SNS, the affectionate emotions of the past decreased." Respondents rate these statements on a scale, commonly ranging from "strongly disagree" to "strongly agree." The specific measurement items and corresponding references of all constructs can be found in Table A1.

Prior to deployment, the questionnaire underwent a rigorous review by two information systems researchers to address content relevance and question ambiguity. Moreover, a pilot test was conducted to examine the clarity, comprehension, and applicability of the survey items. This preliminary study involved 20 participants, representative of our target population. The participants were requested to provide feedback regarding the understandability, relevance, and any ambiguity related to the survey questions. Based on their feedback, minor adjustments were made to improve the wording and sequencing of certain questions to enhance clarity and coherence.

## Results

This study validated the measurement model and the structural model by using the partial least squares structural equation modeling (PLS-SEM) through SmartPLS 4^82^. PLS-SEM also offers some benefits in terms of fewer restrictions on sample size and residuals compared to covariance-based SEM such as AMOS and LISREL^83,84^.

### Common method bias (CMB)

To assess the potential issue of CMB, Harman’s one-factor test was employed^85^. In the exploratory factor analysis, results indicated that the first factor explained 32.801% of the variance, which was considerably less than the threshold of 50%, suggesting that CMB was not a predominant concern in our data. Moreover, in observing the variance inflation factor (VIF) from our regression analysis, all values were well below the critical value of 10, providing further evidence against significant multicollinearity and CMB^86^.

### Measurement model

This study assessed the reliability and validity of the measurement model. This research examined Cronbach's alpha and composite reliability (CR: rho_A, rho_C) to evaluate reliability. If Cronbach's alpha is over 0.60^87^ and CR is greater than 0.7^88^, reliability is achieved. As shown in Table 3, Cronbach’s alpha and CR scores of all the constructs, except for social influence, exceeded the recommended value. Nevertheless, this study decided to retain social influence as other estimates such as CR (rho_C) and AVE were well above the recommended threshold (0.824 and 0.702, respectively).

**Table 3:** Scale reliabilities (95% confidence interval).

Construct	Items	Mean	St. dev	Factor loading	Cronbach's Alpha	CR (rho_A)	CR (rho_C)	Ave
Positive affect	POA1	1.908	0.794	0.855	0.859 [0.819, 0.891]	0.871 [0.839, 0.902]	0.914 [0.892, 0.932]	0.780 [0.735, 0.821]
	POA2	2.248	0.889	0.917				
	POA3	1.986	0.808	0.876				
Negative affect	NEA1	1.819	0.820	0.947	0.944 [0.921, 0.963]	0.944 [0.924, 0.969]	0.964 [0.950, 0.976]	0.899 [0.864, 0.930]
	NEA2	1.780	0.782	0.964				
	NEA3	1.869	0.892	0.933				
Social influence	SOI1	3.553	1.031	0.904	0.588 [0.454, 0.691]	0.646 [0.508, 0.866]	0.824 [0.769, 0.864]	0.702 [0.631, 0.761]
	SOI2	3.447	1.003	0.765				
Flow	FLW1	2.461	0.945	0.922	0.932 [0.913, 0.948]	0.933 [0.916, 0.951]	0.957 [0.945, 0.967]	0.881 [0.851, 0.906]
	FLW2	2.454	0.945	0.956				
	FLW3	2.401	0.922	0.938				
Perceived enjoyment	PEN1	3.376	0.763	0.932	0.926 [0.899, 0.947]	0.928 [0.903, 0.949]	0.953 [0.937, 0.966]	0.871 [0.832, 0.904]
	PEN2	3.429	0.756	0.932				
	PEN3	3.330	0.777	0.936				
Habit	HAB1	3.709	1.028	0.824	0.839 [0.794, 0.874]	0.852 [0.814, 0.888]	0.903 [0.878, 0.923]	0.756 [0.707, 0.799]
	HAB2	3.365	1.148	0.874				
	HAB3	3.560	1.037	0.908				
Addiction	ADD1	2.652	1.179	0.940	0.736 [0.652, 0.803]	1.014 [0.769, 2.669]	0.871 [0.804, 0.907]	0.774 [0.683, 0.830]
	ADD2	2.791	1.128	0.824				

This study investigated convergent validity and discriminant validity to evaluate the validity. Convergent validity was confirmed by investigating both the average variance extraction (AVE) and the factor loads of the items associated with each construct. AVE values ranged between 0.702 and 0.899 which are higher than the expected threshold of 0.5^88^. Factor loadings ranged from 0.765 to 0.964 and are all statistically significant at the *p* = 0.001 levels, supporting that the model has a satisfactory level of convergent validity^89^.

Discriminant validity ensures that a construct is indeed distinct from other constructs by empirical standards^88^. For the present study, two criteria were utilized to determine discriminant validity. Firstly, the square root of the average variance extracted (AVE) for each construct was compared against its correlations with other constructs. As presented in Table 4, the diagonal values (which are the square root of the AVE) for each construct are greater than the off-diagonal values in their respective rows and columns. This demonstrates that the constructs share more variance with their indicators than they do with any other construct, thus meeting the criteria recommended by Fornell and Larcker^88^.

**Table 4:** Correlation matrix and discriminant assessment^88^.

Construct	1	2	3	4	5	6	7
1. Positive Affect	0.883						
2. Negative Affect	0.383	0.948					
3. Social Influence	0.328	0.133	0.838				
4. Flow	0.477	0.344	0.241	0.939			
5. Perceived enjoyment	0.460	− 0.113	0.355	0.280	0.933		
6. Habit	0.308	0.063	0.394	0.296	0.547	0.870	
7. Addiction	0.281	0.279	0.243	0.277	0.132	0.265	0.880

Diagonal values are the square root of AVE.

Additionally, the heterotrait-monotrait ratio of correlations (HTMT) was utilized to assess discriminant validity. As suggested by the literature, an HTMT value below 0.85 indicates the presence of discriminant validity^90^. Table 5 provides the HTMT ratios for all constructs. It's clear from the values that all the ratios are below the 0.85 threshold, further confirming the discriminant validity of our constructs. In conclusion, both the square root of AVE and HTMT criteria confirm the discriminant validity of the constructs utilized in this study.

**Table 5:** HTMT.

Construct	1	2	3	4	5	6	7
1. Positive affect							
2. Negative affect	0.430						
3. Social influence	0.459	0.170					
4. Flow	0.532	0.366	0.320				
5. Perceived enjoyment	0.511	0.123	0.467	0.301			
6. Habit	0.345	0.072	0.538	0.327	0.612		
7. Addiction	0.343	0.341	0.339	0.296	0.140	0.315	

Moreover, HTMT assessment was carried out using bootstrapping with 5000 samples. All 95% confidence intervals substantially veer away from the null value of 1, thereby attesting to discriminant validity (the most elevated value observed is 0.713 between perceived enjoyment and habit; all other pairs showcase values of 0.691 or lower). At the 99% confidence intervals, the upper boundary for the pair perceived enjoyment-habit increases to 0.741, with the subsequent highest value being 0.729.

### Structural model

**Figure 2:**
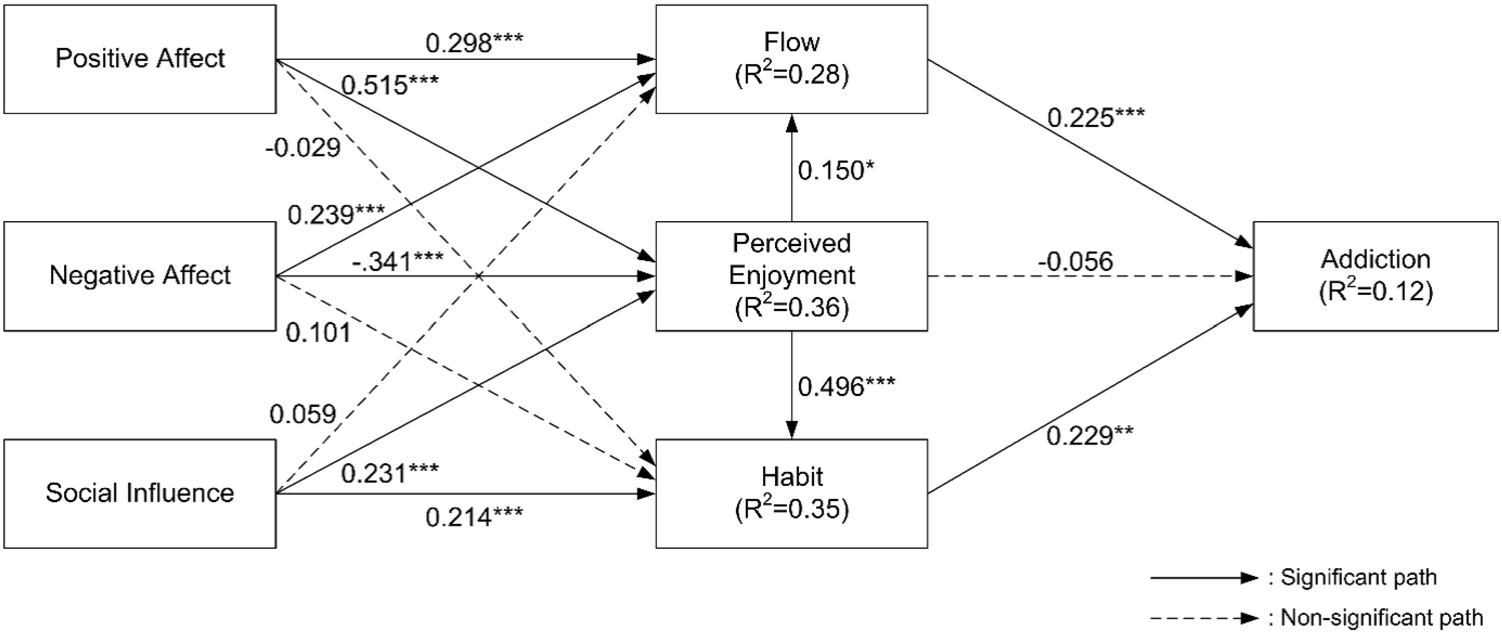
Analysis results.

The hypotheses were tested by using the PLS-SEM technique. This study carried out bootstrapping resampling method with 5000 re-samples. Ten of the fourteen hypotheses in the research framework are supported. Figure 2 shows the analysis results.

As proposed, positive affect has a significant positive influence on both flow (b = 0.298, t = 3.709) and perceived enjoyment (b = 0.515, t = 11.56), strongly supporting H1a and H1b. Contrary to expectations, positive affect does not impact habit (b = − 0.029, t = 0.435), failing to confirm H1c. As anticipated, negative affect has a significant correlation with both flow (b = 0.239, t = 3.456) and perceived enjoyment (b = − 0.341, t = 6.907), thereby supporting H2a and H2b. Conversely, negative affect does not have an impact on habit (b = 0.101, t = 1.592), failing to support H2c. Contrary to expectations, social influence does not correlate with the flow (b = 0.059, t = 1.017), and thus, H3a is not supported. In line with the hypothesis, social influence significantly influences both perceived enjoyment (b = 0.231, t = 3.759) and habit (b = 0.214, t = 3.75), which validates H3b and H3c. As expected, flow is significantly associated with addiction (b = 0.225, t = 3.643), confirming H4. Perceived enjoyment, as hypothesized, exerts a significant positive effect on both flow (b = 0.15, t = 2.476) and habit (b = 0.496, t = 8.199), robustly supporting H5a and H5b. Contradictory to expectations, perceived enjoyment does not influence addiction (b = − 0.056, t = 0.675), failing to confirm H5c. In line with the hypothesis, habit is significantly linked to addiction (b = 0.229, t = 3.09), thereby supporting H6. Overall, the conceptual framework accounted for approximately 11.6% of the variance in addiction. Table 6 shows the summary of the SEM results.

**Table 6:** The results of Hypothesis Testing.

H	Cause	Effect	Coefficient	T-value	Hypothesis
H1a	Positive affect	Flow	0.298	3.709	Supported
H1b	Positive affect	Perceived enjoyment	0.515	11.560	Supported
H1c	Positive affect	Habit	− 0.029	0.435	Not supported
H2a	Negative affect	Flow	0.239	3.456	Supported
H2b	Negative affect	Perceived enjoyment	− 0.341	6.907	Supported
H2c	Negative affect	Habit	0.101	1.592	Not supported
H3a	Social influence	Flow	0.059	1.017	Not supported
H3b	Social influence	Perceived enjoyment	0.231	3.759	Supported
H3c	Social influence	Habit	0.214	3.750	Supported
H4	Flow	Addiction	0.225	3.643	Supported
H5a	Perceived enjoyment	Flow	0.150	2.476	Supported
H5b	Perceived enjoyment	Habit	0.496	8.199	Supported
H5c	Perceived enjoyment	Addiction	− 0.056	0.675	Not supported
H6	Habit	Addiction	0.229	3.090	Supported

The effect size (f^2^) provides an estimation of the magnitude of the difference or the effect of one variable on another. Cohen^91^ suggests guidelines for assessing the size of f^2^ effects: small (0.02), medium (0.15), and large (0.35). Upon evaluation, the computed f^2^ values for our relationships range from 0.001 to 0.320. Specifically, the influence of positive affect on perceived enjoyment exhibits a large effect size (f^2^ = 0.320). The impacts of positive affect on flow and habit show small effect sizes, with f^2^ values of 0.072 and 0.001 respectively. The effects of negative affect on perceived enjoyment, flow, and habit are 0.154, 0.059, and 0.012 respectively, indicating small to medium effect sizes. The influences of social influence on perceived enjoyment, flow, and habit have f^2^ values of 0.074, 0.004, and 0.059 respectively, all suggesting small effect sizes. Lastly, the effects of perceived enjoyment on flow, habit, and addiction are 0.020, 0.244, and 0.002 respectively. Additionally, flow and habit on addiction have effect sizes of 0.051 and 0.040 respectively. These too suggest small to medium effect sizes. It is evident that not all f^2^ values meet the recommended thresholds. The low effect size in certain relationships may suggest that other unconsidered variables or intricate interplays may be at work, which are not captured by the current model. Further research might be necessary to delve deeper into these findings.

The Q^2^ value is an indicator of the predictive relevance of the endogenous latent variables in the model^92^. In PLS-SEM, a Q^2^ value greater than zero indicates that the exogenous constructs have predictive relevance for the endogenous constructs^92^. In assessing the model's predictive power, perceived Enjoyment revealed a Q^2^ value of 0.341, aligned with RMSE and MAE values of 0.819 and 0.634, signifying moderate predictive capacity. Flow has a Q^2^ of 0.245 with RMSE and MAE values at 0.876 and 0.698, indicating modest predictive relevance. Habit demonstrates a Q^2^ of 0.174, associated with RMSE and MAE metrics of 0.917 and 0.707, underscoring satisfactory predictive accuracy. However, addiction records a Q^2^ of 0.089 with RMSE and MAE at 0.961 and 0.804, suggesting a limited predictive scope in the present configuration.

### Ethical approval

This study was approved by an institutional review board of HJ Institute of Technology and Management.

### Informed consent

Informed consent was obtained from all individual participants included in the study.

### Consent to participate

Consent to participate was obtained from all individual participants included in the study.

## Discussion

Our investigation has unearthed certain significant insights that illuminate unique facets of SNS behavior and provide nuanced interpretations that expand upon the extant literature.

The analysis revealed that positive affect influences flow and perceived enjoyment, which aligns with previous research suggesting a significant relationship between positive affect and flow^65,66^, and perceived enjoyment^13^. This suggests that when users are in a better mood, they tend to immerse themselves more in social networking and enjoy it more. However, although Weyland et al. asserted a significant link between positive affect and habit^93^, this study found no such influence. The discrepancy between this study and Weyland's could be explained by the suggestion that positive affect allows users to use SNS consciously to some degree, due to the self-regulation improving characteristics of positive affect after ego depletion^94^.

Similarly, the results indicated that negative affect impacts flow and perceived enjoyment, echoing the findings of prior research^13,95^. This implies that users who experience negative moods are more likely to immerse themselves in SNS while deriving less enjoyment from it. Contrary to expectations, negative affect did not influence habit, suggesting that negative affect does not contribute to the formation of habitual use. It seems that negative emotions, such as distress and aversive mood states, do not necessarily lead to the development of habitual SNS usage. A plausible inference could be that users experiencing negative affect might engage with SNS more consciously or irregularly, trying to alleviate negative moods through certain activities or interactions. This conscious, irregular usage pattern might not provide the consistent reinforcement needed to form a habit. Furthermore, it is also possible that individuals experiencing negative affect might be more likely to engage in a broader range of coping mechanisms, not limited to SNS usage, which would dilute the potential for habit formation on SNS. Lastly, this finding could imply that negative affect might result in less pleasurable or satisfying SNS experiences, which are essential for habit formation. Since habits are often formed when an activity provides consistent satisfaction or reward, if negative affect reduces the perceived reward from using SNS, it could potentially inhibit habit formation.

The findings underscored that social influence significantly affects perceived enjoyment and habit, corroborating previous studies^96–99^. This suggests that users who are more influenced by their social environment find more enjoyment in SNS and use it more habitually. Yet, the absence of a direct relationship with flow offers a fresh dimension: flow, an inherently personal psychological state, appears to be insulated from the sway of social influences. In the context of SNS usage, this might infer that while social influences can shape the manner in which a user engages with the platform (e.g., frequency of use, types of activities participated in), it doesn't necessarily dictate the depth of immersion or the quality of the user's engagement. A user might be influenced by their social circle to use an SNS platform or adopt certain behaviors on it, but whether they achieve a state of flow while using the platform could be more dependent on individual factors such as personal interests, the appeal of the platform's features to the user, or their mood at the time of use. This finding underscores the need for further research to fully understand the interplay between social influence and flow in the context of SNS usage, particularly exploring the influence of individual user characteristics and motivations.

Our study confirmed that flow influences addiction, aligning with existing literature^32,33,62^, suggesting that a higher level of user immersion could lead to greater addiction. The findings could be understood through the framework of how flow experiences can engender repeated and escalated use of a platform, potentially leading to compulsive and addictive behaviors. In the context of SNS usage, a flow state might occur when users are so absorbed in browsing posts, interacting with others, or creating content that they lose track of time and are oblivious to external distractions. When users frequently experience flow while using an SNS platform, it can lead to higher levels of satisfaction and enjoyment. These positive experiences can, in turn, motivate them to seek out repeated instances of such experiences, potentially leading to more frequent and prolonged use of the platform. Over time, this escalated use can develop into habit formation, and in some cases, escalate into addiction. This is particularly likely if the SNS platform becomes a primary source of positive reinforcement or pleasure for the user, or if the user becomes reliant on the platform to escape negative feelings or realities.

The study found that perceived enjoyment impacts flow and habit, which is consistent with previous studies^10,100^. This implies that users who derive more enjoyment from SNS are more likely to immerse themselves in its usage and develop a habit. However, contrary to some previous research^9,76,77^, our findings showed that perceived enjoyment is not related to addiction, suggesting that factors other than perceived enjoyment might have a more significant impact on SNS addiction.

Finally, our analysis revealed that habit significantly impacts addiction, in agreement with prior findings^10,30^. This suggests that habitual SNS users are more prone to developing an addiction. Habit may be represented by frequent checking of notifications, constant scrolling of feeds, or habitual posting of updates, among other behaviors. When these behaviors become ingrained, they form a pattern that requires minimal cognitive effort to initiate. The repeated engagement with the SNS platform may then become an automatic response to certain triggers, such as a notification sound or even a moment of boredom. In the early stages, these habits may simply represent high engagement with the platform. However, over time and with continued repetition, these habitual behaviors can lead to dependence and eventually addiction. This is particularly likely when the habitual use of the platform is paired with strong positive reinforcement (e.g., receiving likes or comments) or serves as a coping mechanism for negative emotions. Addiction, in this context, is characterized by an inability to control SNS usage despite negative consequences, a preoccupation with the SNS platform, and potential distress or withdrawal symptoms when access to the platform is denied or limited. Thus, the development of strong, unconscious SNS usage habits could significantly contribute to the onset of such addictive behaviors.

## Conclusion

### Summary

This study illuminated the precursors of SNS addiction, highlighting flow, habits, and perceived enjoyment as pivotal determinants. The research suggested that positive affect, negative affect, and social influence were central contributors to the onset of addiction and its precursors. A survey of SNS users was carried out, and the PLS-SEM methodology was employed to evaluate both the measurement and structural models. The findings demonstrated that positive affect significantly impacted both flow and perceived enjoyment, while negative affect notably influenced flow and perceived enjoyment. Additionally, social influence significantly affected perceived enjoyment and habit. The results underscored that perceived enjoyment influenced addiction significantly through both flow and habit.

### Theoretical implications

This study presents several significant academic contributions. Firstly, it enriches the SNS literature by introducing a comprehensive model that incorporates flow, habit, and perceived enjoyment to explain SNS addiction among users. While previous studies on SNS addiction have primarily focused on health, loneliness, and attachment^17,52,101^, our research intricately weaves these concepts into a coherent framework, shedding light on their combined and individual roles in driving SNS addiction. The cascading effect delineated in this research, tracing the journey from perceived enjoyment to addiction through the intricate interplay of flow and habituation, heralds a groundbreaking perspective in SNS addiction studies. This view not only challenges the dominant linear frameworks often seen in the literature but also introduces a much-needed nuanced understanding. The triangulated perspective brought forth in this work goes beyond merely linking variables; it unravels the depth of their interconnections, offering scholars a comprehensive lens that is markedly distinct from the fragmented approaches seen in prior works. Its novelty lies in its multi-dimensional approach, capturing the essence of intertwined variables rather than isolating them. For scholars, this presents an invitation to delve deeper into understanding the complexities of SNS addiction, and, more broadly, prompts a reconsideration of how other behavioral addictions might be driven by a synergy of factors rather than linear paths. Such insights pave the way for richer, more detailed investigations in the future, shaping the trajectory of addiction research.

Secondly, this research takes a notable step further by integrating a dualistic scale of users’ affect—both positive and negative—into the fabric of our understanding of SNS addiction. This bifurcation in affective states offers a fresh lens to comprehend the intricacies of user engagement with SNS platforms. A particularly arresting observation from our study is the role of these affective dimensions in modulating the 'flow' within SNS engagements. Both positive and negative affects emerged as potent enhancers of flow. This illuminates an intriguing facet of user behavior: irrespective of whether users are in a state of contentment or grappling with emotional discomfort, they exhibit a heightened propensity to lose themselves in the labyrinth of SNS activities. The underlying reasons for this phenomenon could be multifaceted. Users might be navigating the SNS realm in search of vicarious contentment by witnessing the seemingly euphoric lives of their peers, especially during their own moments of distress. Alternatively, some might be on a quest to find peers who portray lives perceived as less joyous, perhaps as a means to assuage their own emotional tumult. Yet another possibility is the unintentional immersion in SNS activities as a coping mechanism against feelings of relative deprivation. It's noteworthy that while these affective states profoundly influence flow, they remain inconsequential in forming habits, suggesting a discernible boundary between transient emotions and ingrained behaviors. The differential impact of these emotional scales on perceived enjoyment, aligning with the theoretical assertions of their orthogonal relationship^25^, further enriches our comprehension of the complex tapestry of SNS engagement dynamics.

Lastly, this study probes the role of social influence in the process of addiction formation. Given that the unique function of SNS is fostering social relationships, the mutual influence among users can significantly impact SNS activity. Yet, the analysis shows that social influence does not directly precipitate addiction or flow. This indicates that intrinsic motivations might be stronger than extrinsic factors in fostering addiction and flow. This finding provides a useful perspective for future studies on SNS addiction, suggesting a systematic classification of internal and external explanatory factors. Social influence significantly affects habits and perceived enjoyment, indicating that users are more likely to use SNS habitually and enjoy it more when they frequently hear about it from people around them. However, addiction and flow seem to require a higher level of concentration than mere habit formation. Typically, SNS users engage with the platforms for light hobbies or social interactions, rather than work or academic purposes. Against this backdrop, it appears that even when influenced by their surroundings, SNS users primarily maintain a habitual level of engagement.

### Practical implications

This paper presents various practical implications. Firstly, habit is a primary factor in SNS addiction. Thus, users should be guided to avoid excessive SNS usage to prevent addiction. Strategies like limiting usage or curtailing frequent app access can aid addicted individuals^102^. Secondly, the study underscores perceived enjoyment's significant impact on flow and habit. SNS providers might intersperse public interest messages within feeds to reduce habit formation and potential addiction. Thirdly, the data reveals positive affect's substantial role in addiction, flow, and enjoyment. It's vital for developers to balance positive affect's enhancement of user experience without inducing addiction. Fourthly, despite negative feelings, users engage with SNS. Understanding their motives can help service providers foster healthier interactions and mitigate negative emotions. Lastly, the influence of social word-of-mouth bolsters habits and enjoyment in SNS. Providers can amplify engagement by incentivizing users to share platform benefits.

### Limitation and further research

Despite several contributions, this study also has some limitations. First, the study was conducted exclusively in South Korea, limiting its geographical scope. SNS addiction could potentially vary according to culture, nationality, and the maturity of online social networks across different nations. Therefore, future research could enhance the generalizability of the results by investigating user behaviors relating to addiction in various countries. Second, this paper examined SNS addiction solely among Facebook users. However, user engagement and addiction behavior might differ based on the platform's structure or operation methods. Consequently, future studies should consider obtaining samples from users of other platforms such as Pinterest, Twitter, YouTube, and Instagram. This would allow for a deeper exploration of how user addictions form, in relation to the distinct characteristics of each SNS.

### Supplementary Information

Supplementary Information.

## Data Availability

The datasets used and/or analyzed during the current study available from the corresponding author on reasonable request.
