# Supplementary material for: Predictors of social networking service addiction
Source: Sci Rep. 2023 Oct 4;13:16705. doi: 10.1038/s41598-023-43796-2 (PMC10550908; doi:10.1038/s41598-023-43796-2)
Supplement: Supplementary file 1 — Supplementary Information. [file 41598_2023_43796_MOESM1_ESM.docx]

# Appendix A

**Table A1.** List of Model Constructs and Items

| Construct | Items | Mean | Source |
| --- | --- | --- | --- |
| Positive  Affect | POA1 | I get excited when I use SNS. | [Beatty and Ferrell (1998)](#_ENREF_5) |
|  | POA2 | I am passionate while using SNS. |  |
|  | POA3 | I am proud while using SNS. |  |
| Negative  Affect | NEA1 | I suffer while using SNS. | [Beatty and Ferrell (1998)](#_ENREF_5) |
|  | NEA2 | I get angry while using SNS. |  |
|  | NEA3 | I feel annoyed while using SNS. |  |
| Social Influence | SOI1 | If I don't use SNS, I seem out of date. | [Li (2011)](#_ENREF_44) |
|  | SOI2 | People or the media recommend SNS to me. |  |
| Flow | FLW1 | While using SNS sites, my attention was focused solely on SNS. | [Gong et al. (2020)](#_ENREF_20) |
|  | FLW2 | I was completely focused while using social media sites. |  |
|  | FLW3 | I was deeply immersed in social media while using social media sites. |  |
| Perceived Enjoyment | PEN1 | It is fun to use SNS. | [Davis et al. (1992)](#_ENREF_16) |
|  | PEN2 | Using SNS is interesting. |  |
|  | PEN3 | Using SNS gives me pleasure. |  |
| Habit | HAB1 | I use SNS to kill time. | [Limayem et al. (2007)](#_ENREF_45) |
|  | HAB2 | I habitually use SNS whenever I have spare time. |  |
|  | HAB3 | I use SNS to relieve boredom. |  |
| Addiction | ADD1 | I was immersed in SNS and experienced a decrease in conversations when meeting people. | [Osatuyi and Turel (2018)](#_ENREF_48) |
|  | ADD2 | As I used SNS, the affectionate emotions of the past decreased. |  |
